# Impact of a program to prevent incivility towards and assault of healthcare staff in an ophtalmological emergency unit: study protocol for the PREVURGO On/Off trial

**DOI:** 10.1186/1472-6963-14-221

**Published:** 2014-05-19

**Authors:** Sandrine Touzet, Pierre-Loïc Cornut, Jean-Baptiste Fassier, Marie-Annick Le Pogam, Carole Burillon, Antoine Duclos

**Affiliations:** 1 Hospices Civils de Lyon, Pôle Information Médicale Évaluation Recherche, Lyon F-69003, France; 2 Université de Lyon, EA Santé-Individu-Société 4128, Lyon F-69002, France; 3 Hospices Civils de Lyon, Hôpital Edouard Herriot, service d’ophtalmologie, Lyon F-69003, France; 4 Hospices civils de Lyon, Service de médecine et santé au travail, Lyon F-69003, France; 5 Université de Lyon, UMR T 9405, Lyon F-69373, France; 6 Institute of Social and Preventive Medicine, University Hospital of Lausanne, Lausanne CH-1010, Switzerland

**Keywords:** Emergency unit, Ophthalmology, Workplace violence, Healthcare workers, Violence, Assault, Alternating time series design

## Abstract

**Background:**

The emergency department has been identified as an area within the health care sector with the highest reports of violence. The best way to control violence is to prevent it before it becomes an issue. Ideally, to prevent violent episodes we should eliminate all triggers of frustration and violence. Our study aims to assess the impact of a quality improvement multi-faceted program aiming at preventing incivility and violence against healthcare professionals working at the ophthalmological emergency department of a teaching hospital.

**Methods/Design:**

This study is a single-center prospective, controlled time-series study with an alternate-month design. The prevention program is based on the successive implementation of five complementary interventions: a) an organizational approach with a standardized triage algorithm and patient waiting number screen, b) an environmental approach with clear signage of the premises, c) an educational approach with informational videos for patients and accompanying persons in waiting rooms, d) a human approach with a mediator in waiting rooms and e) a security approach with surveillance cameras linked to the hospital security. The primary outcome is the rate of incivility or violence by patients, or those accompanying them against healthcare staff. All patients admitted to the ophthalmological emergency department, and those accompanying them, will be enrolled. In all, 45,260 patients will be included in over a 24-month period. The unit analysis will be the patient admitted to the emergency department. Data analysis will be blinded to allocation, but due to the nature of the intervention, physicians and patients will not be blinded.

**Discussion:**

The strengths of this study include the active solicitation of event reporting, that this is a prospective study and that the study enables assessment of each of the interventions that make up the program. The challenge lies in identifying effective interventions, adapting them to the context of care in an emergency department, and thoroughly assessing their efficacy with a high level of proof.

The study has been registered as a cRCT at clinicaltrials.gov (identifier: NCT02015884).

## Background

### An increase in violence at hospitals and more specifically at emergency units

The National Institute for Occupational Safety and Health has long recognized violence as a workplace hazard for health care workers [1]. The emergency department (ED) and treatment rooms are among the most frequent locations for violent events to take place in the health care setting. Around the world, the ED has been identified as an area within the health care sector with the highest reports of violence [2,3]. In the United Kingdom, it was found that 50% of attacks against health care workers occurred in the ED [4]. High rates of workplace violence have also been experienced by ED nurses in the United States of America [2], in Australia [5] and in Ireland [6]. The current rate of violence towards staff in the ED is reported to be 2.0 to 2.8 incidents per 1,000 patients, and is rising [7,8]. However, most of the violent episodes in the ED go unreported [8].

In France, the National Observatory of Violence in Healthcare Facilities (Observatoire National des Violences en milieu de santé) received 11,344 reports of violence (against people or property) in 2012 from 352 hospitals. This is nearly double the number of reports compared to 2011 (in part due to the higher number of reporting facilities). Among all reported violence, the vast majority, 85%, involve personal aggressions [9]. Teaching hospitals were amongst the facilities most affected, as were EDs (14% of reports). Those responsible for reported violence were generally patients (73%), or those accompanying them (28% of the violence in the EDs), whereas hospital staff were the victims in 77% of cases. Those working at healthcare facilities are particularly exposed to the risk of workplace violence [10,11].

### Types and consequences of workplace violence

When attempting to describe violent behavior, three levels of aggressiveness are distinguished by order of severity: incivility, which results from a lack of respect for others and manifests itself as relatively harmless acts, verbal abuse or physical threat (insults, threatening behavior) and physically violent acts [12]. This incivility/violence can have repercussions on the physical and/or emotional health of the victims, and thus on their well-being and the quality of their work. Staff have been shown to suffer emotional symptoms similar to post-traumatic stress disorder, job dissatisfaction, and early feelings of burnout, while hospitals suffer the financial burden of decreased productivity and excessive lawsuits [4,5,13-19].

### Precipitators of violence and aggression

Different factors, such as anxiety, boredom, lack of information, lack of understanding of triage times and categories, and waiting times, may influence violent behavior [2,5,20,21]. While patients themselves are frequently the main aggressor, relatives and visitors are also known to instigate violence.

The best way to control violence is to prevent it before it becomes an issue. Ideally, to prevent violent episodes we should eliminate all triggers of frustration and violence.

### Objective

The study aims to assess the impact of a violence prevention program geared to reducing the incivilities or violence against health care workers in an ophthalmological emergency department (OED) at a teaching hospital. Our prevention program was designed in order to minimize the discomfort of patients visiting the OED.

## Methods

### Study design

**Figure 1:**
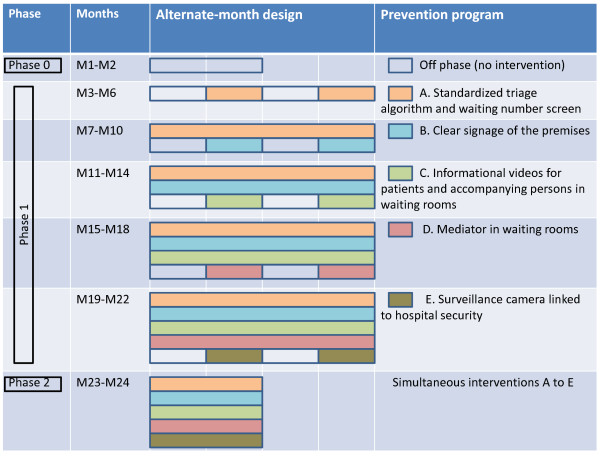
**The multifaceted intervention.** This figure presents the different components of the multifaceted intervention, as well as the alternate-month design.

This quasi-experimental study is a single-center prospective, controlled time-series study with an alternate-month design (On and Off periods). The prevention program combines 5 complementary interventions that are implemented step by step (interventions A to E, phase 1 - see Figure 1). Each intervention has no residual effect during Off periods and is independent of the others. Each intervention is based on a design that alternate two one-month On (intervention) and Off (control) periods over a total of four months. After the 4-month On/Off period, each intervention becomes permanent alongside the previously implemented interventions.

There are two 2-month observational phases: a “pre-intervention” phase (phase 0) and a “simultaneous interventions A to E” phase (phase 2) see Figure 1.

### Setting

This study takes place at an adult OED, at a university teaching hospital over a 24-month period. It is open 24 hours a day, 7 days a week and handles all types of medical and surgical ophthalmological emergencies. The unit treats approximately 22,600 patients per year, i.e. an average of 62 patients a day.

### Participants

#### Patients and those accompanying them

All patients registering at the OED are included in the study. Those accompanying the patient (family, friends, etc.) are also enrolled.

#### Health care professionals and administrative staff

The OED team (7 nurses, 6 ward aides, 2 orthoptics students, 7 residents in ophthalmology and 4 senior ophthalmologists) operating on a rotating schedule to provide care 24/7 are enrolled in the study. The OED team present during a week day is composed of 4 nurses, 4 ward aides, 2 orthoptics students, 1 or 2 residents in ophthalmology, and 1 on-call senior ophthalmologist. Four admitting clerks are also enrolled.

### Intervention

This program is based on the successive implementation of five complementary interventions (Interventions A to E).

#### Intervention A (organizational approach): standardized triage algorithm and patient waiting number screen

The first intervention will be based on the implementation of a standardized computer algorithm for patient triage as soon as they arrive in the unit. This enables the degree of urgency of care to be calculated, the order in which the patient is to be seen by a medical doctor to be specified and related medical procedures to be started. The unit has developed its own algorithm specifying 6 levels of severity from lowest to highest. The first level indicates a non-emergency situation whereas level 6 designates the need for immediate care. Triage is performed at reception by a nurse trained on the algorithm, which will enable unit staff to know how many patients are waiting for care, the level of severity for each patient, and length of time each has been waiting.

A patient waiting number will be based on the triage algorithm. This number and the consultation room will be displayed on a video screen in the waiting room when the patient is called. Nursing staff will come to the waiting room to get patients who cannot read the screen (as they used to do before implementation of the algorithm and patient waiting number screen).

This algorithm will involve both informatics technology (IT) revisions and staff process/culture changes to address the admission process. IT revisions will include integrated interfaces between the OED computer system (admission, triage, OED record) with the hospital computer system [22].

#### Intervention B (environmental approach): clear signage of the premises

Appropriate signage may be a factor for the prevention of aggressive behavior and insistent questions. We will create new signage to help patients to understand where to go between the hospital admission desk, the waiting rooms, the nurse office and the consultation rooms. The sequence varies depending on the day of the week (week or weekend) and the time of day (daytime or night time).

#### Intervention C (educational approach): informational videos for patients and accompanying persons in waiting rooms

There will be video screens in each waiting room displaying information for patients and accompanying persons, i.e. profile of medical staff, average number of consultation per day, rules for provision of care and the average patient waiting time, as well as general-interest educational information on eye diseases. This enables patients to understand the reason for their wait and makes the wait more bearable.

#### Intervention D (human approach): mediator in waiting rooms

There will be a mediator in the waiting rooms in order to liaise between the patients and those accompanying them and the medical staff in the event of a problem. This mediator will intervene in order to deal with any uncivil or aggressive behavior, and to prevent an escalation to violence as well as to reduce tension in the unit. Their role will also involve identifying individuals presenting a risk in order to quickly intervene in the event of a minor incident so as to prevent the situation from deteriorating.

#### Intervention E (security approach): surveillance camera linked to hospital security

The final intervention will involve installing surveillance cameras throughout the unit (admissions desk, waiting rooms, corridors, and consultation rooms) linked to the hospital security post. These cameras will be signposted. The fact that these cameras are signposted enables a dissuasive approach to the prevention of uncivil or violent behavior.

### Control group

No intervention liable to affect reception of patients, the organization of care or the practices of health care professionals will be implemented during the Off phases. Patients will be seen based on the order of severity as assessed by the physician and based on the reason for the consultation recorded at admission.

### Outcomes and measurements

#### Primary outcome

The primary outcome is professional-reported incivilities or violence by patients, or those accompanying them, against health care staff at the OED or against other patients and/or those accompanying them. Health care professionals will be informed of the study and trained to notify any incivility or violence by a patient they may be subject to or witness.

Incivility or violence are to be described using a classification that distinguishes four levels, from least to most severe (see Table 1), based on the French National Observatory of Violence in Hospitals [9].

**Table 1:** Four levels of Incivility or violence, from least to most severe


Level 1	Insistent questions, incivility, rudeness, occupation of the corridor, spitting, making noise (telephone, etc.)
Level 2	Insult or verbal abuse without threat
Level 3	Verbal abuse or physical threat
Level 4	Intentional violence, assault, vandalism or damage to equipment

#### Secondary outcomes

The secondary outcomes are

OED waiting times, measured as the period of time between the patient’s administrative check-in and the care of a physician.

The patient leave rate measured as the proportion of patients having left the OED without having received medical treatment,

Work-related stress of OED health care personnel, as measured by the Karasek questionnaire, a French validated questionnaire,

Satisfaction of patients treated at the OED.

### Sample size calculation

The number of patients has been calculated based on following hypotheses:

A 20% initial rate of incivility/violence against OED staff, whatever the severity of the violence,

A minimum 2.5% reduction in incivility/violence with each new intervention A to E,

An average of 62 patients per day expected,

The independence of the 5 interventions,

A risk alpha of 5% and the statistical power of 80%.

The statistical unit is the patient admitted to the OED. Using the Casagrande and Pike method [23], and based on the aforementioned hypotheses, the sample size required is 30,224 patients for the 5 alternating intervention phases (see Table 2).

**Table 2:** Sample size required by intervention phase

**Interventions**	**TControl**	**TIntervention**	**Sample size required**
Intervention A	20%	17,5%	7802
Intervention B	17,5%	15%	6986
Intervention C	15%	12,5%	6108
Intervention D	12,5%	10%	5166
Intervention E	10%	7,5%	4162

Note:

TControl is the rate of incivility/violence in the control group (Off).

TIntervention is the rate of incivility/violence in the intervention group (On).

### Blinding

Health care providers, participants and researchers will not be blinded to the intervention phase.

### Ethical approval and informed consent

Approval for the study was obtained from the hospital ethics committee, from the Sud Est III Institutional Review Board (study identifier: 2008–036 B) and from the French Data Protection Agency (CNIL).

As the promoter of this biomedical research, which falls within the scope of French Law n°2004-806 of 9 August 2004, the Hospices Civils de Lyon has acquired liability insurance coverage and stood as guarantors of the proper execution of the study.

Consent of the health care staff was sought before the study. As stipulated by French law, and given the study methodology and type of intervention, no patient consent was required [24].

### Data analysis

Data analysis will be performed by the data management and analysis center, using SAS/STAT® software. The unit of analysis will be the patient admitted to the OED. Analyses will be performed on an intent-to-treat basis.

Analyses of data from phase 1 “Implementation of interventions A to E”, will enable estimation and comparison of the average rates of violence committed against OED staff between the On and Off periods of each intervention (primary analysis). It will also enable the average rate of violence against patients, the mean rate of patients’ leaving before care, and average OED waiting time to be calculated for each intervention (secondary analyses). Finally, we will attempt to identify predictive factors for the primary outcome and secondary outcomes. Measurements of the incivility/violence rates in the intervention and controls groups will be compared for each of the 5 intervention phases using a Khi-2 test. The average waiting times in the intervention/control groups will be compared for each of the 5 interventions using a Student test.

Analyses of data from phase 0 “pre-intervention” and phase 2 “Simultaneous interventions A to E” will enable the proportion of admitted patients committing violence against medical staff to be compared before and after implementation the overall prevention program (primary analysis) and healthcare staff well-being and OED patient satisfaction to be compared.

The rate of incivility and other outcome criteria (e.g. mean waiting times, patient satisfaction, etc.) will be compared before and after implementation of the prevention program (phase 2 vs phase 0) using standard tests.

A bivariate analysis will select potential confounding factors with a view to multivariate modeling.

Modeling to compare primary and secondary outcome criteria between phase 0 and phase 2 will use nonlinear and linear mixed models (proc GLIMMIX and proc MIXED of SAS/STAT® software v9.2), taking into account confounding factors and fixed and random effects.

### Time frame

The planned Inclusion period is 24 months:

“Pre-intervention” phase (phase 0) for 2 months. During this period, no specific intervention will implemented as part of the study. In all, around 3,780 patients will be enrolled over two months.

“Implementation of interventions A to E” phase (phase 1) for 20 months. In all, 37,820 patients will be enrolled.

“Simultaneous interventions A to E” phase (phase 2) for 2 months. This final observational period will enable us to compare outcome criteria before and after implementation of the prevention program. In all, around 3,780 patients will be enrolled over two months.

Furthermore, this study includes an approximately 6-month preparation and healthcare staff awareness campaign on reporting incivility and violence.

### Trial status

The “Pre-intervention” phase (phase 0) is currently underway (January and February 2014). Patient enrollment began in January 2014. Data is currently being collected. No statistical processing has been performed on the primary and secondary outcome criteria.

A patient satisfaction survey was carried out in June. The questionnaire results are being kept at the data management and analysis center.

## Discussion

### Discussion of study design

The strengths of this study include active solicitation of event reporting, the fact that this is a prospective study and the study design enabling assessment of each intervention in the program.

In certain situations, it may be of interest to use an alternate-month study design, i.e. alternating On (intervention) and Off (control) periods which provide a series of before and after studies with interventions repeated over time [25]. This type of study design can be used only when there is no residual affect during Off periods. It is particularly well-suited to assess the impact of IT tools [26]. This design may be of particular interest when assessing the impact of an intervention in a single health care facility, which makes a randomized controlled study difficult to perform [27].

Unlike a simple before/after study, this design offers the advantage of providing a control group that enables more thorough assessment of the impact of a repeated intervention at a health care facility.

Moreover, in addition to the overall effect, the effect of each of the 5 interventions can be assessed. It nonetheless requires the event measured to be sufficiently frequent during each alternating period.

### Discussion of primary outcome criterion

All of the healthcare staff at the OED were trained to report acts of incivility/violence for each patient and/or relative coming to the unit. A number of scales exist to measure violence, such as the Overt aggression scale [28]. We created a scale based on the findings of the French National Observatory of Hospital violence [9]. Unlike other studies, which consider only physical violence, our scale also takes into account verbal abuse and rudeness. Although verbal abuse is often not included [29], most studies consistently show that verbal abuse, threats and assaults are common [13,30,31].

However, perceptions of what constitutes ‘abuse’, ‘threats’ or ‘assault’ may be less clear, which may have an impact on the reporting and subsequent handling of such incidents [32]. The under-reporting of incivility/violence is a well-known phenomenon, namely when reports are made directly to the hospital administration [18,32-35]. Lack of time is the main reason for under-reporting given by healthcare professionals. The fact that incivility often goes unrecognized, due to habit and understanding of/empathy for patients, is also mentioned. In order to curb this under-reporting, healthcare professionals were trained using virtual cases (short clinical vignettes) in order to provide all staff enrolled in the study with a shared definition of incivility or violence (see Table 1). In addition, any act of incivility/violence toward healthcare care staff or other patients was recorded in the patient’s medical record, simultaneously alongside other essential medical information.

### Discussion of the intervention

Several levels of interventions are possible: interventions at the patient level, interventions at the level of the healthcare professional facing acts of incivility/violence, and interventions regarding security [36]. Interventions regarding healthcare professionals aim to enable medical staff to recognize signs of potentially violent situations and to know how to prevent the escalation of violence [37,38]. Although educational initiatives on managing patient aggression may assist in improving staff confidence and perception of safety, there are few data to prove that these programs actually reduce the number of incidents and their consequences in the long term [15,39,40]. Security interventions involve checking of patients and interventions of the police [41,42]. In accord with the staff of the OED, we chose to develop primarily patient-centered actions in order to minimize patient discomfort during their visit to the OED.

There are a number of different theories of violence [8,20,21]. The Frustration—Aggression theory is one of these, defined by a lack of understanding of the surrounding environment, which leads to frustration and then aggression. Our intervention is multifaceted, combining interventions on various dimensions that may influence the behavior of patients and/or those accompanying them, based on the Frustration-Aggression theory. The intervention was designed by a working group involving the different healthcare professional of the OED. Four types of approaches were selected: organizational, environmental, interpersonal, and security (video surveillance cameras). Our measures, which primarily target the experience and behavior of the patients, include implementation of a series of organizational and functional measures such as improving the conditions of patient admission and stays in the OED (cleanliness of the premises, clear signage, etc.), reduced waiting times (standardized triage and orientation procedure by admissions nurses), mediators in relation with waiting patients and those accompanying them, and security measures (video surveillance cameras and maintaining patients in the waiting room).

Since the 1990s, overcrowding of emergency units has been a problem affecting all developed countries [43]. This phenomenon occurs when demand for emergency care exceeds a unit’s capacity to provide care within a reasonable waiting time. In France, overcrowding of emergency units has increased considerably over the last 20 years with an average increase of 5% per year. According to the Centers for Disease Control and Prevention, two-thirds of US metropolitan EDs experience overcrowding [44]. This overcrowding, which previously affected mainly general emergency departments, now also affects OEDs. This flow of patients responsible for longer waiting times has led to a high level of patient dissatisfaction. This frequently results in administrative complaints, patients leaving before receiving care, and verbal or physical abuse of healthcare professionals. Waiting time is an indirect measurement of crowding, which is a contributing factor of violence [5,18,45].

Waiting time is a well-known primary factor of patient dissatisfaction, as a result leading to incivility and violence against healthcare staff. By optimizing the order in which patients are treated (prioritization algorithm with call-up screen), we wish to reduce this waiting time and thus curb incivility by patients and/or those accompanying them [4]. As well, this waiting time must be as bearable as possible and video messages (information on the OED and educational messages) should help to pass the waiting time and reduce the feeling of being abandoned in the waiting room.

The use of a triage algorithm should help to optimally prioritize patients for care. The principal of triage is defined as a dynamic decision-making process performed at patient admission in order to specify an order of priority for care [46]. This algorithm is integrated in the IT system, which includes administrative check-in of patients when they arrive at the OED, the recording of data from the medical records and the prioritization of each patient in terms of OE. This type of tool has been shown as useful in reducing the number of patients that leave before being seen by a physician [22] and in reducing waiting times [43,47]. This tool involves informatics work, with the cooperation of the hospital IT department, and the creation of an algorithm including all of the medical information potentially collected by the nurses at the OED admissions desk. The triage algorithm was design by physicians from the OED participating in the study. This work was based on the current French recommendations. Nurses are to be trained to use this tool. It is nurses who, in the OED studied, receive the patient once they have completed admissions procedures with the hospital administrative desk.

In a study published in Science, Keizer et al. examine the influence of environmental factors on the occurrence of deviant behavior in society [48]. Likewise, in the OED, we can suppose that a well-organized, clean, well-lit waiting room is one factor in preventing incivility/violence against healthcare staff.

We will use a mediator whose role is to prevent the escalation of violence. De-escalation is a gradual resolution of a potentially violent and/or aggressive situation through the use of verbal and physical expressions of empathy, alliance and non-confrontational limit setting. However, nurses often require specialized training in this respect-based technique [20]. In short, de-escalation involves defusing, negotiation and conflict resolution, [49] with the aim of recognizing signs of impending violence and preventing it before it happens [50]. While this technique is useful for minimizing violent behavior, not all nurses are trained to use these techniques [49]. We have thus decided to entrust an external, specially trained third party with this task.

Workplace violence has emerged as a significant problem compromising security, self-esteem, work performance, relationships, and overall health of ED employees. There is a paucity of large, well-designed studies supporting any strategy aimed at preventing ED workplace violence [36].

## Abbreviations

ED: Emergency department; OED: Ophthalmological emergency department; IT: Informatics technology.

## Competing interests

The authors declare that they have no competing interests.

## Authors’ contributions

The study was conceptualised and designed by ST, PLC, MALP and AD. PLC and CB are the Co-Chief Investigators, provided leadership for the project. ST, PLC, JBF and AD contributed to the development of the intervention. MALP and AD planned the statistical analysis. ST drafted the manuscript. All authors reviewed the draft version, made suggestions and approved the final version.

## Pre-publication history

The pre-publication history for this paper can be accessed here:

http://www.biomedcentral.com/1472-6963/14/221/prepub

## The PREVURGO study group

Nassira Amamra, Emmanuelle Aubert, Sylvain Beccat, Daniel Betito, Laetitia Bouveret, Dominique Delaunay, Pauline Occelli, Philippe Sarnin and Sylvie Sullerot
